# Modern and traditional cooking methods affect the antioxidant activity and phenolic compounds content of *Trachystemon Orientalis* (L.) G. Don

**DOI:** 10.1371/journal.pone.0299037

**Published:** 2024-02-23

**Authors:** Yagmur Demirel Ozbek, Ozlem Saral, Perim Fatma Turker

**Affiliations:** 1 Department of Nutrition and Dietetics, Faculty of Health Sciences, Recep Tayyip Erdoğan University, Rize, Turkiye; 2 Department of Nutrition and Dietetics, Faculty of Health Sciences, Başkent University, Ankara, Turkiye; National Research Centre, EGYPT

## Abstract

*Trachystemon orientalis* (L.) G. Don is a medicinal plant with beneficial effects on human health. Its antioxidant and phenolic compound content is higher than most natural plants. This is the first study on the cooking of this consumed plant. This study investigated how different cooking methods and times affect the antioxidant activity and phenolic compound content of *Trachystemon orientalis* (L.) G. Don. The Folin-Ciocalteu method (FCR), ferric-reducing antioxidant power (FRAP), copper-reducing antioxidant capacity (CUPRAC), and 2,2-diphenyl-1-picrylhydrazyl (DPPH) radical scavenging activity were used to evaluate the antioxidant activity and total phenolic content (TPC). Phenolic compounds were also determined by high-performance liquid chromatography (HPLC). Microwave cooking, stir-frying and sous vide increased TPC and antioxidant activity (p<0.05). Steaming decreased TPC and antioxidant activity (p<0.05). It was determined that the best cooking method and time was stir-frying for 15 minutes (TPC, CUPRAC and FRAP values 45.18±3.91 mg GAE/g DW, 15559.39±106.90 mmol Troloks/g DW and 555.10±24.05 μmol Fe (II)/g DW, respectively). Raw *Trachystemon orientalis* (L.) G. Don was detected with caffeic acid (31.53±0.25 mg/100 g DW). New phenolic compounds (protocatechuic acid and p-coumaric acid) were formed by boiling, stir-frying, microwaving, and sous vide methods. In conclusion, regarding antioxidant activity and phenolic compounds of *Trachystemon orientalis* (L.) G. Don; the best cooking methods are microwave, stir-frying, and sous vide (p<0.05). The most wrong cooking method is steaming (p<0.05).

## Introduction

*Trachystemon orientalis* (L.) G. Don belongs to the Boraginaceae family, consisting of 100 genera and 2000 species. This plant is distributed in the Black Sea region, Eastern Bulgaria, Western Caucasus, and various habitats in Turkey [1]. Members of this species are 30–40 cm tall, perennial hairy plants with rhizome root structures, broad leaves, and blue-red flowers. According to the Turkish Flora records, *Trachystemon orientalis* (L.) G. Don is widely distributed in moist, shady, dense forests and riversides at altitudes of 50 to 1000 m [2].

Studies on the plant have determined that it is important in preventing cancer and is antidiabetic [3, 4]. It was also determined to have antioxidant, antifungal and herbicide properties [1, 5]. In addition to being very rich in phenolic compounds, the plant was also found to have higher antioxidant properties than asparagus and broccoli when the antioxidant activity was examined [6]. This plant is cooked and consumed in various regions of the Black Sea, stems and leaves of *Trachystemon orientalis* (L.) G. Don can be consumed together or separately. Leaves are boiled in water or fried in oil. Also, pickles are made from the stems [7].

Cooking is the process carried out to make foods suitable for consumption, increase their flavor, and make them digestible [8]. Boiling, steaming and stir-frying are considered traditional cooking methods [9]. However, the utilization of sous vide and microwave cooking techniques is increasing day by day with technological changes [10–12]. It is considered that the plant’s physical structure changes with cooking methods and its nutritional value. It has been determined that cooking methods and different temperatures and periods may directly affect the nutritional value of the plant as well as the antioxidants [13].

Antioxidants have been proven to have an essential role in preventing cancer, cardiovascular diseases, diabetes, neurological diseases and immune system diseases by clearing reactive species in the body [14]. Phenolic compounds are natural antioxidants. The most important function of phenolic compounds in plants is to take an active role in the defense mechanism of plants [15]. Phenolic compounds are structures synthesized during plant development that resist infection, injury and UV radiation [16]. Antioxidant activity and phenolic compound content of plants have been the subject of many studies for years. These phenolic compounds have several advantages for human health, such as anticancer, anti-inflammatory, anti-snake, and antimicrobial effects [16, 17]. However, in recent years, the effect of cooking on antioxidant activity and phenolic compound content has only recently begun to be examined [8, 18–20].

The study aims to investigate the effect of different cooking methods on the antioxidant activity and phenolic compound content of *Trachystemon orientalis* (L.) G. Don.

## Materials and methods

### Chemicals

Acetonitrile, methanol, ethanol, sodium hydroxide, sodium acetate, glacial acetic acid, iron (III) chloride, hydrochloric acid, H2SO4, and sodium carbonate were purchased from Merck (Germany). Gallic acid, protocatechuic acid, ferulic acid, rutin, catechin, benzoic acid, chlorogenic acid, o-coumaric acid, vanillic acid, quercetin, caffeic acid, t-cinnamic acid, epicatechin, kaempferol, p-coumaric acid, 2,2-diphenyl-1-picrylhydrazyl (DPPH-) stable radical, neocuproine (2,9-dimethyl-1,10-phenanthroline), 6-Hydroxy-2,5,7,8-tetramethylchroman-2-carboxylic acid (Trolox) were obtained from Sigma-Aldrich Co. (USA). Folin-Ciocalteu’s phenol reagent and 2,4,6-tri(2-pyridyl)-S-triazine (TPTZ) were obtained from Fluka Chemie GmbH (Switzerland). Ultrapure water (Sortorius, Arium® 611, Germany) was used for the preparation of all chemical solutions.

### Preparation of *Trachystemon Orientalis* (L.) G. Don

*Trachystemon orientalis* (L.) G. Don was obtained from Pazarköyü (41° 10’ 35’’ north; 40° 33’ 25’’ east) in the center of Rize, on the Eastern Black Sea coast of Turkey. Pazarköyü is located 261 meters above sea level. Approximately 2 kg of plant samples were harvested in May 2021. *Trachystemon orientalis* (L.) G. Don was cleaned immediately after harvesting by separating the leaves and stems from the roots. They were washed thoroughly under water and dried with a paper towel. Leaves and stems were used for cooking. Roots were not used since they were not consumed. The plant was cut into homogeneous pieces (3x3 cm) with a knife and weighed 100 g each. Samples were used fresh without preservation.

This study was part of a PhD study at the University of Baskent (Ankara, Turkey). This study was approved by the Baskent University Institutional Review Board (Project no: KA21/216).

#### Cooking methods

**Fig 1:**
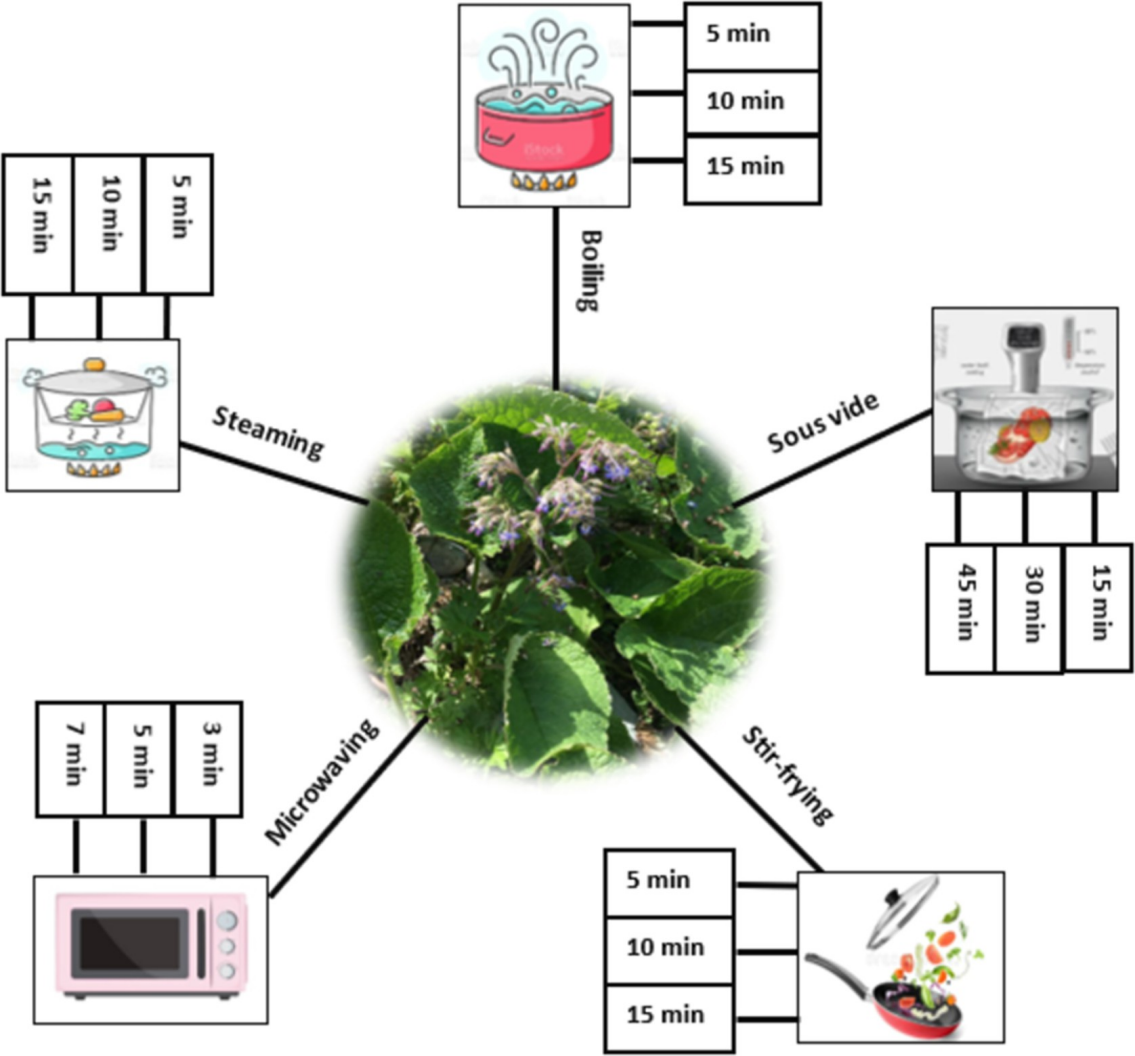
Different cooking methods applied on *Trachystemon orientalis* (L.) G. Don.

*Trachystemon orientalis* (L.) G. Don was subjected to five different cooking methods: boiling, stir-frying, microwaving, steaming, and sous vide. For each cooking method, three different cooking times (boiling (5, 10 and 15 min); steaming (5, 10 and 15 min); stir-frying (5, 10 and 15 min); microwaving (3, 5 and 7 min); sous vide (15, 30 and 45 min)) were experimented. Previous studies [11, 18, 21–24] were taken as examples in determining these cooking methods and times. Then, local cooking techniques were observed and the researchers conducted preliminary experiments. The cooking times of *Trachystemon orientalis* (L.) G. Don plants, which were evaluated as rare, medium-cooked, and overcooked, were selected, and the times to be followed for cooking were determined (Fig 1). One hundred g of *Trachystemon orientalis* (L.) G. Don was used for three repetitions for each cooking method and time. Three samples were preserved raw.

*Boiling method*. Fresh plant samples were added to 400 mL of boiling (97.3±0.5°C) pure water in three identical steel pots (15 cm diameter).

*Steaming method*. Plant samples were cooked by boiling the material in pure water at atmospheric pressure (97.3±0.5°C) in a covered steamer.

*Stir-frying method*. Plant samples were cooked in a Teflon pan at 180°C with 10 mL sunflower oil.

*Microwaving method*. The samples were placed on glass plates with 10 mL of distilled water and cooked in a microwave oven set to 600 W.

*Sous vide method*. The samples were packaged using a vacuum packaging machine (Electrola EVM-42). The packaged plant samples were cooked at 80°C using sous-vide cooking equipment (PolyScience SousVide Professional Classic Series).

After all cooking processes were finalized, the samples were cooled to room temperature.

#### Preparation of the extracts

Raw and cooked samples were homogenized in a blender (Schafer—Germany) for 2 minutes. For all analyses, 50 mL of methanol was mixed with approximately 10 g of raw and cooked homogenized samples. The samples were left to digest with a mechanical shaker for one day. The solution was filtered through Whatman No.1 filter paper. The clear extracts were utilized to determine phenolic content and antioxidant activity.

Since calculations are based on dry matter, 3–4 g of raw or cooked homogenized samples were dried in an oven at 70°C for at least two days until reaching constant weight [18].

#### Determination of the total phenolic content

Total phenolic content was determined using the Folin-Ciocalteu method developed by Slinkard and Singleton. [25]. Briefly, 0.4 mL 0.5 N Folin-Ciocalteu reagent was mixed with 20 μL extract and 680 μL distilled water. After 3 min, 0.4 mL of 10% sodium carbonate solution was added. The mixture was then incubated in the dark for 2 hours at room temperature. The absorbance of the samples was recorded at 760 nm on a UV-VIS spectrophotometer (Spectro UV-VIS Double PC-8 autocell, LabomedInc, Culver City, USA). Gallic Acid (GA) was adopted as a reference, and the results were expressed as milligram GA equivalent per gram of dry matter (mg GAE/g dry matter).

#### Determination of antioxidant activity

The ferric-reducing antioxidant power (FRAP) method was carried out according to Benzie & Strain [26]. Preparation of FRAP reagent, 200 mL 300 mM acetate buffer (pH 3.6): 20 mL 10 mM 2,4,6-tri(2-pyridyl)-s-triazine (TPTZ) solution: 20 mL of 20 mM FeCl_3_ solution. TPTZ and FeCl_3_ solutions were prepared fresh every day. The FRAP reagent was kept at 37°C in a water bath before analysis. Finally, 100 uL of extract and 3 mL of FRAP reagent were mixed, and the reaction mixture was incubated at 37°C for 4 min. The method is based on reducing the Fe^+3^—TPTZ complex to blue-colored Fe^+2^—TPTZ in the presence of antioxidants by reading the absorbance at 593 nm. Standard solutions of FeSO_4_ .7H_2_O were used to generate a standard curve. Results are expressed as μmol Fe(II)/g dry matter.

Copper-reducing antioxidant capacity (CUPRAC) was evaluated according to the method developed by Apak et al. [27]. The method was carried out by adding 10 mM CuCl_2_, 10 mM CuCl_2_, ammonium acetate buffer (1 M, pH 7.0) and 7.5 mM neocuproine solution (1 mL of each) to a tube. Then, 0.2 mL of extract and 0.9 mL of deionized water were mixed. After one hour, the absorbance was measured at 450 nm using a UV-VIS Spectrometer. Antioxidant activity was determined according to the Trolox standard, and the results were expressed as mmol Trolox/g dry matter.

2,2-diphenyl-1-picrylhydrazyl (DPPH•) radical scavenging activity was determined by modifying the method developed by Yu et al. [28]. In the study, a methanolic solution of DPPH• radical was prepared as 4 mg/100 ml. Samples and Troloks® standards were prepared at different concentrations. 750 μL of DPPH• solution was added to 750 μL of extracts of varying concentrations. After one hour incubation, absorbance was measured at 517 nm. The results were determined at 50% inhibited DPPH concentration and expressed as IC_50_. The lower the IC_50_ value, the higher the DPPH• scavenging value.

#### Determination of phenolic compounds

In order to determine the phenolic compound content of the plant, the method employed by Akyüz et al. [29]. In this method, the methanol of the plant extracts was evaporated to dryness under reduced pressure in a rotary evaporator (Buchi R-100 Rotary Evaporator, Switzerland) at 50°C. The residue was then dissolved with 10 mL of dislodged water. This was preceded by liquid-liquid extraction with 5 mL diethyl ether and 5 mL ethyl acetate thrice. The organic phases were combined in the same flask and evaporated to dryness under reduced pressure in a rotary evaporator at 40°C. The residues were weighed and dissolved in 5 mL methanol for HPLC-UV analysis (Thermo Finnigan, San Jose, CA, USA).

HPLC-UV analyses of phenolic compounds were performed on a reversed-phase C18 column (4.6 x 150 mm inner diameter, 5 μm particles; Fortis France) with a gradient program with two solvent systems (A: 70% acetonitrile in methanol, B: 2% acetic acid in distilled water). The elution gradient was performed from high polarity and low pH to low polarity and high pH. Gradient: 0–3 min 5% B; 3–8 min 5–15% B; 8–10 min 15–20% B; 10–12 min 20–25% B; 12–20 min 25–40% B; 20–30 min 40–80% B. The injection volume was 25 μL, and the column temperature was regulated at 30°C in the column oven. A 1.2 mL/min flow rate was utilized, and detection was performed at 280 and 315 nm. In this system, 15 phenolic compounds (gallic acid, protocatechuic acid, catechin, epicatechin, ferulic acid, p-coumaric acid, o-coumaric acid, rutin, vanillic acid, chlorogenic acid, benzoic acid, caffeic acid, quercetin, t-cinnamic acid, kaempferol) were analyzed simultaneously at 280 and 315 nm. The separated compounds were identified by comparing the retention times of the peaks in the chromatogram obtained with the retention times of standards previously analyzed under the same operating conditions.

Research does not involve human subjects or animal experiments; therefore, ethics committee approval was not obtained.

### Statistical analysis

Results are expressed as mean value ± standard deviation (SD). The data obtained were analyzed with the SPSS software package version 23.0 (SPSS Inc. Chicago, IL, USA). One-way analysis of variance (ANOVA) and Duncan’s multiple range test procedure were utilized to test for significant differences between different group means. Pearson correlation analysis was employed to measure the relationship between two variables.

## Results and discussion

### Total phenolic content and antioxidant activity

Total phenolic content values of raw and cooked *Trachystemon orientalis* (L.) G. Don plants are presented in Table 1. The mean total phenolic content of raw *Trachystemon orientalis* (L.) G. Don was 4.83±0.32 mg GAE/g DW. Total phenolic contents of *Trachystemon orientalis* (L.) G. Don samples collected from Düzce and Sinop at any time of spring were reported to be in the range of 0.75±0.32–472.22±5.50 mg GAE/g DW [4, 5, 30]. This difference in total phenolic content may have been due to conditions such as soil structure, sun intake, climate characteristics, and harvest time of the plant [31]. These findings are within the expected range.

**Table 1:** Effect of different cooking methods on total phenolic content (TPC) and antioxidant activity of *Trachystemon orientalis* (L.) G. Don.

Cooking Method	Cooking time (min)	TPC (mg GAE/ g DW)	CUPRAC (mmol Troloks/g DW)	FRAP (μmol Fe (II)/g DW)
**Raw**	-	4.83±0.32^h^	138.86±5.17^g^	44.74±0.59^ı^
**Boiling**	5	6.92±0.47^gh^	83.38±1.02^h^	39.73±1.84^ı^
	10	5.15±0.38^h^	105.85±1.63^gh^	31.51±0.78^j^
	15	9.17±0.17^f^	134.43±0.90^g^	53.76±1.60^h^
**Steaming**	5	2.33±0.10^ı^	56.35±2.33^h^	19.63±0.24^k^
	10	0.05±0.02^j^	10.38±1.39^ı^	5.95±0.14^l^
	15	0.06±0.03^j^	11.31±0.18^ı^	7.60±0.62^l^
**Stir-frying**	5	14.83±1.73^e^	440.39±8.66^c^	148.20±1.99^d^
	10	14.76±2.47^e^	317.63±13.57^d^	127.91±5.40^e^
	15	45.18±3.91^a^	15559.39±106.90^a^	555.10±24.05^a^
**Microwaving**	3	11.25±0.66^f^	288.57±22.14^de^	87.42±5.50^g^
	5	9.12±1.09^fg^	323.39±44.56^d^	106.97±5.76^f^
	7	28.37±2.40^b^	742.52±18.52^b^	260.61±10.46^b^
**Sous vide**	15	15.20±1.76^e^	218.07±2.02^f^	110.75±3.44^f^
	30	17.81±0.98^d^	238.43±2.83^f^	139.91±3.60^d^
	45	21.87±1.27^c^	254.94±1.69^e^	175.08±2.24^a^

^a^There is no significant difference between groups with the same letters (a-l) in the same column.

Plant tissues contain many different compounds with antioxidant activity. It is difficult to measure all antioxidant elements. Therefore, different methods have been developed to quantify antioxidant activity [12]. This study utilized CUPRAC, FRAP and DPPH methods to measure antioxidant activity. Free radical scavenging activity for DPPH radical was expressed as IC_50_. There is an inverse relationship between IC_50_ and antioxidant activity in all samples.

**Fig 2:**
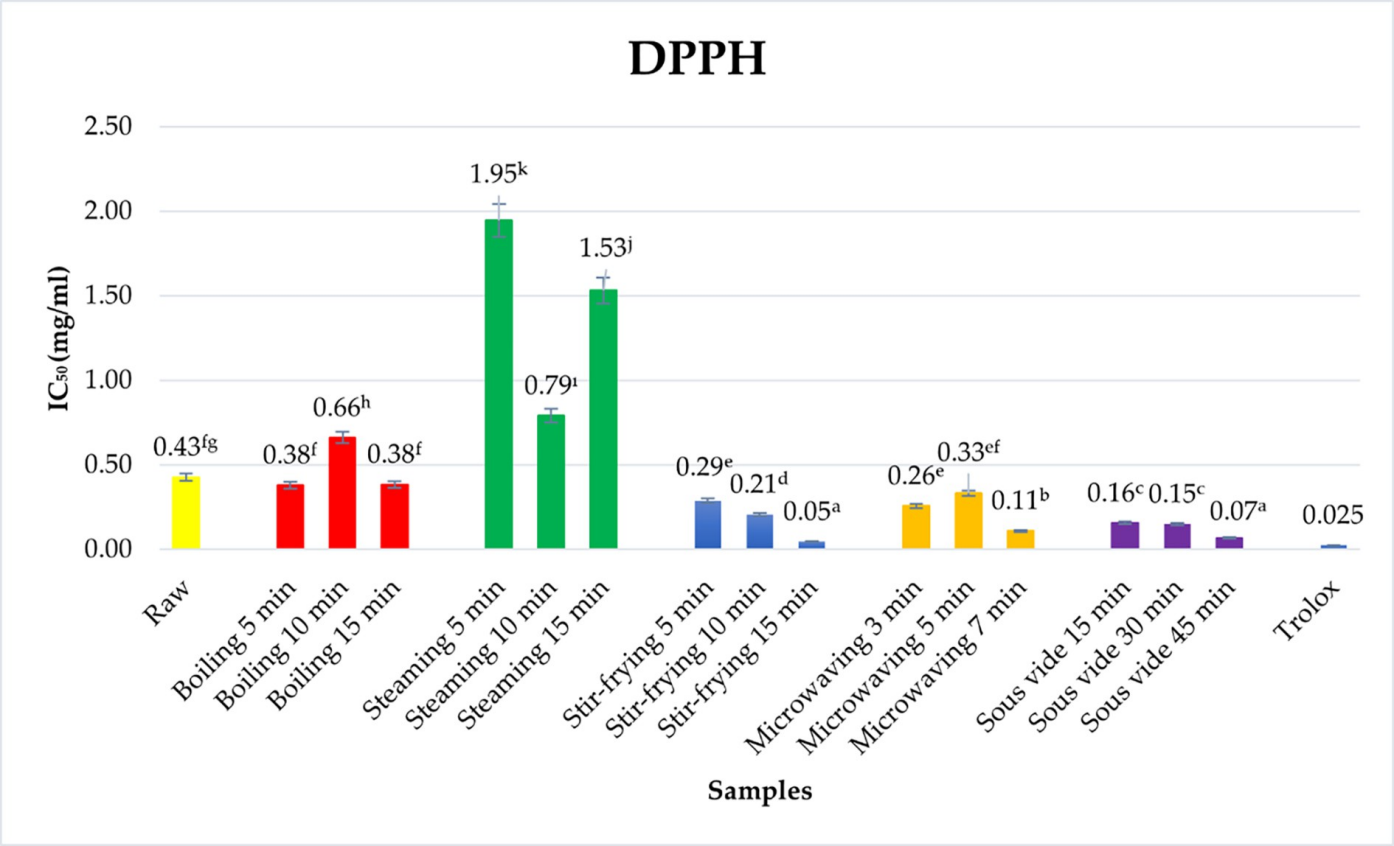
Effect of different cooking methods on DPPH• radical scavenging activity of *Trachystemon orientalis* (L.) G. Don.

CUPRAC and FRAP values of raw and cooked *Trachystemon orientalis* (L.) G. Don plants are provided in Table 1, and DPPH values are presented in Fig 2. When the antioxidant activity of *Trachystemon orientalis* (L.) G. Don was evaluated, and it was observed that the CUPRAC value was 138.86±5.17 mmol trolox/g DW, the FRAP value was 44.74±0.59 μmol Fe(II)/g DW, and DPPH value was 0.43±0.33 mg/mL. When CUPRAC, FRAP and DPPH values were examined in many studies, it was found that the antioxidant activity of raw *Trachystemon orientalis* (L.) G. Don plant was higher than many plants in nature [32–37]. The average CUPRAC values of *Hypericum montbretii*, *Paliurus spina-christi Mil* and *Hypericum bupleuroides* were determined to be 1.07±0.14–0.19±0.05 mmol trolox/g DW [32]. A study was conducted with Pistachio (*Pistacia vera* L.), Knotgrass (*Celtis aetnensis*), Turpentine Tree (*Pistacia terebinthus* L.), Hawthorn (*Crataegus monogyna*), Sumac (*Rhus Coriaria* L.), Almond (*Prunus dulcis*), Bitter Almond (*Amygdalus Amara*), and Fennel (*Foeniculum Vulgare*) plants cultivated in Şırnak province and medicinally used by the people. As a result of the study, it was observed that the CUPRAC value was in the range of 3.90±0.45–0.07±0.00 mmol trolox/g DW and the FRAP value was in the range of 0.79±0.28–0.02±0.00 μmol Fe(II)/g DW [33]. In the study conducted by Ceylan et al. Giant fennel (*Ferula communis* L.), Rumex (*Rumex patientia* L.), Acanthus (*Gundelia tournefortii* L.), Isgin (*Rheum ribes* L.), Asphodelus (*Asphodeline taurica*), Polygonum (*Polygonum arenastrum*), Chives (*Allium schoenoprasum* L.), Ferula (*Ferula orientalis* L.) plants were observed to have CUPRAC values between 54.41±3.64–0.53±0.13 mmol trolox/g DW and FRAP values between 42.50±2.44–0.31±0.03 μmol Fe(II)/g DW [34]. The IC_50_ values of the leaves of amaranth, solanum and cucurbita plants consumed in Uganda are in the range of 0.56–1.48 mg/ml [34]. Antioxidant activity varies with plant variety, genotype, geographical region and living conditions [38].

Total phenolic content and antioxidant activity of *Trachystemon orientalis* (L.) G. Don, according to cooking methods, is provided in Table 1. There was no significant difference in total phenolic content and antioxidant activity in boiling (5 and 10 min), while it was observed to increase in prolonged boiling (15 min) (p<0.05). Total phenolic content and antioxidant activity were found to increase in stir-frying, sous vide, and microwave cooking. There was a decrease in steam cooking (p<0.05). When compared in terms of duration, it was detected that total phenolic content and antioxidant activity increased in *Trachystemon orientalis* (L.) G. Don boiling, microwaving, stir-frying, and sous vide cooking methods. On the contrary, total phenolic content and antioxidant activity decreased with the prolonged cooking time. In the literature, no study investigates the antioxidant activity of *Trachystemon orientalis* (L.) G. Don plant about cooking.

Total phenolic content and antioxidant activity increased due to boiling in artichokes and green leafy plants specific to India, which were boiled for a long time (10 minutes) [39, 40]. In boiled Chinese cabbage, total phenolic content and antioxidant activity also increased with the prolongation of the boiling time [41]. The cell wall’s softening with the effect of heat in boiling and the release of phenolic compounds and antioxidant elements from the cellular matrix may have been involved [36, 42].

Total phenolic content and antioxidant activity of *Trachystemon orientalis* (L.) G. Don’s cooking in the microwave increased at the 5th minute; however, no significant increase was observed at the 10th minute. After the 10th minute, it exhibited a significant increase (p<0.05). Similarly, different studies on green leafy plants reported that antioxidant activity increased due to microwaving, which increase persisted in long-term cooking [36, 41, 43]. In this study, it was determined that microwaving of the plant was higher in terms of total phenolic content and antioxidant activity than boiling and steaming. Similar results were obtained in similar studies. In a study conducted with cabbage, boiling was significantly lower than microwaving [44]. Microwave cooking of the B. vulgaris plant was determined to have high antioxidant activity compared to steaming and blanching [43]. It may be the case that the structure of the cell wall is disrupted by the effect of rays during microwave cooking, and phenolic compounds are released accordingly [45].

While the total phenolic content and antioxidant activity of the steamed Trachystemon orientalis (L.) G. Don plants were determined to be the lowest compared to other cooking methods; TPC and antioxidant activity of the plant decreased significantly as a result of steaming (p<0.05). Similar to this study, in two studies conducted with cabbage, it was determined that antioxidant activity decreased as a result of steaming [10, 12]. It was observed that the total phenolic content of steamed faba beans decreased, and the decrease increased with the prolongation of time [46]. In the study by Laib and Barkat, it was reported that steaming decreased antioxidant activity and the decrease continued with time [47]. While TPC and antioxidant activity increased in some plants, it decreased in some plants in steam cooking [18]. This difference may be due to plant variety and environmental factors. Since steaming has no contact with the heat source in steaming, the cooking temperature is much lower than that of other cooking methods [48]. For this reason, it is thought that the antioxidant elements released as a result of the softening of the cell walls of some plants are not released. However, despite the low temperature, a loss of sensitive antioxidant elements may have occurred. This explains the low total phenolic content and antioxidant activity.

In this study, the TPC and antioxidant activity of the fried plant increased. However, while this increase in total phenolic content remained until the 10th minute, it lasted with the prolongation of time (p<0.05). It was observed that antioxidant activity increased proportionally with time. Similarly, it was determined that total phenolic content and antioxidant activity increased due to stir-frying tropical plants consumed in Sri Lanka [49]. Şengül et al. reported that while the amount of total phenolic content decreased due to steaming beet, turnip and black radish, it increased as a result of microwaving and stir-frying [50]. Our study determined the highest total phenolic content and antioxidant activity at the end of 15 minutes of stir-frying. The increase in phenolic substances and antioxidant elements may have been observed as a result of stir-frying, color change and Maillard reaction [42]. Depending on the intensity and duration of the heat treatment in stir-frying, the compounds formed as a result of the Maillard reaction vary. These compounds can demonstrate antioxidant properties. At the same time, the decrease in the plant’s water during stir-frying and the penetration of oil may have prepared the ground for new compounds [49].

It was determined that the total phenolic content and antioxidant activity of *Trachystemon orientalis* (L.) G. Don plant cooked with sous vide method increased in a linear manner with time (p<0.05). While the total phenolic content and antioxidant activity of broccoli in which the sous vide method was performed decreased [51], it was reported to increase in a study conducted with carrots [52]. Doniec et al. stated that when boiling and sous vide methods were performed on Brussels sprouts, the total phenolic content was the same in boiling compared to raw but increased in the sous vide method [53]. In a study in which boiling, steaming, sous vide, microwave cooking and stir-frying processes were performed on broccoli, it was determined that the highest antioxidant activity was obtained as a result of sous vide, microwave cooking and stir-frying, respectively [54]. The reason for quite different results in the studies may be the difference in plant species and cooking times [23].

While a few studies argue that different cooking methods increase antioxidant activity [19, 42, 55, 56], a few others claim that cooking decreases plant antioxidant activity [21, 57]. There are different views explaining the increase in antioxidant activity in plants: 1) Antioxidants are released and activated by disruption of the cell wall and cell structure, 2) Increasing the number of antioxidants by increasing the reactions with the effect of heat, 3) The enzymes involved in oxidation reactions lose their activity, and the activity of antioxidants increases with the effect of heat 4) New antioxidants may be released as a result of reactions (Maillard reaction) [20, 44, 58].

### Phenolic compound content

Phenolic compound content of raw and cooked *Trachystemon orientalis* (L.) G. Don plant is presented in Table 2. Phenolic compounds are present in nature. Although phenolic compounds vary depending on genetic factors, they are also affected by geographical and pedoclimatic factors. Phenolics are important bioactive compounds. Phenolic compounds are affected by many factors, including cooking methods and duration [45]. Although there is information about the phenolic content of green leafy vegetables, there is insufficient information about the phenolic compounds of plants belonging to the Boraginaceae family. In studies conducted with the Boraginaceae family, caffeic acid, p-coumaric acid, rosmarinic acid, gallic acid, protocatechuic acid and ferulic acid compounds were identified [59–62].

**Table 2:** Effect of different cooking methods on phenolic compound content of *Trachystemon orientalis* (L.) G. Don.

Cooking Method	Cooking time (min)	PCA (mg / 100 g DW)	CA (mg / 100 g DW)	p-CA (mg / 100 g DW)
**Raw**		-	31.53±0.25^**j**^	-
**Boling**	5	1.71±.0.07^**l**^	19.56±0.88^**k**^	-
	10	4.37±0.18^**j**^	33.47±1.49^**j**^	-
	15	2.81±0.01^**k**^	38.43±1.67^**ı**^	-
**Steaming**	5	-	7.77±0.56^**k**^	-
	10	-	1.01±0.04^**l**^	-
	15	-	1.36±0.04^**l**^	-
**Stir-frying**	5	10.22±1.10^**e**^	175.87±2.00^**f**^	-
	10	18.17±0.14^**c**^	214.22±2.21^**c**^	-
	15	90.90±1.04^**a**^	1107.38±3.47^**a**^	-
**Microwaving**	3	6.65±0.06^**g**^	129.15±2.61^**g**^	-
	5	8.47±0.09^**f**^	179.13±1.99^**e**^	-
	7	21.68±0.37^**b**^	487.65±3.74^**b**^	8.73±0.09^**c**^
**Sous vide**	15	16.63±0.45^**d**^	201.95±3.57^**d**^	22.63±0.35^**a**^
	30	6.07±0.12^g**h**^	82.53±2.11^h^	6.00±0.21^**c**^
	45	5.74±0.15^**hı**^	86.11±2.04^ı^	10.50±0.31^b^

^a^There is no significant difference between groups with the same letters (a-l) in the same column.

^b^PCA: protocatechuic acid, CA: caffeic acid, p-CA: p-coumaric acid

Caffeic acid was detected in the raw state of *Trachystemon orientalis* (L.) G. Don. Cooking resulted in changes in phenolic compounds, and protocatechuic acid, caffeic acid and p-coumaric acid were identified. Protocatechuic acid, caffeic acid and p-coumaric acid are compounds frequently mentioned in the medical field. These phenolic compounds have been reported to have antioxidant, antimicrobial, neuroprotective, anti-inflammatory, analgesic and anti-cancer effects [63–65]. In particular, protocatechuic acid is a phenolic acid frequently used in the medical field and has anti-inflammatory and anti-diabetic effects on human health [66].

Protocatechuic acid was not detected in the raw *Trachystemon orientalis* (L.) G. Don plant was released during boiling, stir-frying, microwaving, and sous vide methods. Especially the protocatechuic acid content of *Trachystemon orientalis* (L.) G. Don plant fried for a long time was observed to be the highest. Protocatechuic acid is also present in some plants, such as chia seeds and saw palmetto, in raw form when extracted with methanol. This suggests that protocatechuic acid may be present raw in some plants while it may be released by cooking in others [67, 68]. Previous studies also determined that protocatechuic acid content increased as a result of cooking [69–71]. Researchers hypothesize that protocatechuic acid exists in bound forms in complex structures with lignins and hydrolyzable tannins, carbohydrates or proteins, so it is released with heat and moves out of the cell.

While *Trachystemon orientalis* (L.) G. Don was determined to contain caffeic acid in raw form; it was found to decrease at the end of the 5th minute during boiling. Caffeic acid increased with the prolongation of time. It decreased significantly in steaming (p<0.05). While an increase in caffeic acid was reported in stir-frying and microwaving, the increase remained with the prolongation of time. On the other hand, in sous vide, an increase was observed in the first cooking time, while a decrease was observed over time (p<0.05). The cooking methods with the highest caffeic acid content were stir-frying and microwaving. Data from many different studies emphasize that caffeic acid varies positively or negatively according to cooking method and time [11, 40, 72–74]. These differences may be due to the structure and content of plant species.

p-coumaric acid was not detected in raw, boiled, steamed and fried Trachystemon orientalis (L.) G. Don. However, it was detected in microwave cooking and sous vide methods. Similar to this study, some studies have reported that an increase in p-coumaric acid was observed in the sous vide method [11, 74, 75]. This supports the idea that new phenolic compounds may be released with cooking [76]. It is believed that closed vacuum cooking in sous vide method may have an important role in the emergence of p-coumaric acid content.

### Correlation of TPC, CUPRAC, FRAP and DPPH

The correlation between total phenolic content, CUPRAC, FRAP and DPPH values of raw and cooked *Trachystemon orientalis* (L.) G. Don plants are presented in Table 3. The correlations between total phenolic content and antioxidant activity measured by CUPRAC, FRAP and DPPH were significant. The relationship was consistent with previous studies on plants [54, 73, 77, 78]. Due to the antioxidant properties of phenolic compounds, it has been possible to provide results compatible with antioxidant activity methods. Heat treatments may trigger the formation of phenolics, which are bioactive compounds, as a result of cooking, which may have an effect on antioxidant activity [76].

**Table 3:** Correlation between antioxidant activity determination method and total phenolic content values in all cooking methods.

		TPC	FRAP	CUPRAC	DPPH•
**Boiling**	**TPC**	**1**			
	**FRAP**	**0.959** **	**1**		
	**CUPRAC**	**0.604** *	**0.667**	**1**	
	**DPPH**•	-0.734*	-0.639	-0.778*	1
**Steaming**	**TPC**	1			
	**FRAP**	0.992**	1		
	**CUPRAC**	0.994**	0.992**	1	
	**DPPH**•	-0.734*	-0.799*	-0.760*	1
**Stir-frying**	**TPC**	1			
	**FRAP**	0.987**	1		
	**CUPRAC**	0.967**	0.990**	1	
	**DPPH**•	-0.902**	-0.903**	-0.878**	1
**Microwaving**	**TPC**	1			
	**FRAP**	0.995**	1		
	**CUPRAC**	0.954**	0.996**	1	
	**DPPH**•	-0.812**	-0.813**	-0.790*	1
**Sous vide**	**TPC**	1			
	**FRAP**	0.898**	1		
	**CUPRAC**	0.904**	0.980**	1	
	**DPPH**•	-0.888**	-0.904**	-0.858**	1

*p<0.05

** p<0.01 TPC: Total phenolic content, FRAP: Ferric reducing antioxidant power, CUPRAC: Copper reducing antioxidant capacity, DPPH: 2,2-diphenyl-1-picrylhydrazyl

In the study, it was determined that there was a correlation between the data obtained from CUPRAC, FRAP and DPPH methods. This may be due to the fact that all three tests are based on similar mechanisms based on electron transfer to reduce oxidant substances. These findings were compatible with many previous studies [12, 79].

## Conclusion

The antioxidant defense system has an important place in protecting against diseases. Phenolic compounds are natural antioxidants. It has been known for years that the antioxidant content of plants varies with cooking method and duration. However, the cooking method and cooking time vary for each plant. This study is the first on the cooking of *Trachystemon orientalis* (L.) G. Don plant. In line with the results obtained in this study, it was observed that the best cooking method in terms of antioxidant activity and phenolic compound content of *Trachystemon orientalis* (L.) G. Don’s plant was stir-fried for a long time. As a result of stir-frying, new antioxidant elements may have been formed with the effect of high heat. For this reason, a data bank can be created in terms of antioxidant activity by finding the best cooking method and times of plants present in nature and consumed. Thus, the scientific world and society can be enlightened about cooking.

## Supporting information

S1 Table TPC and antioxidant activity absorbance values, absorbance mean and standard deviation. (PDF)

S1 Fig TPC and antioxidant activity standard graphs. (PDF)

S2 Fig HPLC chromatogram of all cooking methods. (PDF)
