# Supplementary material for: Modern and traditional cooking methods affect the antioxidant activity and phenolic compounds content of Trachystemon Orientalis (L.) G. Don
Source: PLoS One. 2024 Feb 23;19(2):e0299037. doi: 10.1371/journal.pone.0299037 (PMC10890727; doi:10.1371/journal.pone.0299037)
Supplement: S1 Table — (PDF) [file pone.0299037.s001.pdf]

Total phenolic content absorbance values, absorbance mean and standard deviation

**Cooking Method**	**Cooking time**	**Absorbance 1**	**Absorbance 2**	**Absorbance 3**	**Absorbence mean**	**Standard deviation**
**Raw**		4.562616	4.731810	5.182996	4.8258	0.32
**Boling**	5 **min**	7.345334	7.012798	6.421625	6.9266	0.47
	10 **min**	5.540296	5.13868	4.777225	5.1521	0.38
	15 **min**	9.288916	9.240143	8.971889	9.1670	0.17
**Steaming**	5 **min**	2.417648	2.184341	2.388485	2.3302	0.13
	10 **min**	0.049543	0.075619	0.049543	0.0582	0.02
	15 **min**	0.054909	0.083809	0.02601	0.0549	0.03
**Stir-frying**	5 **min**	15.7903	15.86793	12.84029	14.8328	1.73
	10 **min**	16.10117	16.27343	11.90935	14.7613	2.47
	15 **min**	49.70361	42.78432	43.07262	45.1868	3.91
**Microwaving**	3 **min**	11.68578	11.58624	10.49132	11.2545	0.66
	5 **min**	10.34823	8.244932	8.770756	9.1213	1.09
	7 **min**	29.43901	30.05105	25.61378	28.3679	2.40
**Sous vide**	15 **min**	16.47479	15.97697	13.15594	15.2026	1.79
	30 **min**	18.92596	17.0868	17.43381	17.8155	0.98
	45 **min**	23.24863	21.61298	20.75798	21.8732	1.27

Ferric reducing antioxidant power absorbance values, absorbance mean and standard deviation

**Cooking Method**	**Cooking time**	**Absorbance 1**	**Absorbance 2**	**Absorbance 3**	**Absorbence mean**	**Standard deviation**
**Raw**		44.10200735	45.26806609	44.85161654	44.74	0.59
**Boling**	5 **min**	37.61132	40.63786	40.95262	39.73	1.84
	10 **min**	30.64917	31.71678	32.16162	31.51	0.78
	15 **min**	52.31151	55.48081	53.5	53.76	1.60
**Steaming**	5 **min**	19.7041	19.35955	19.83331	19.63	0.24
	10 **min**	5.988101	5.795558	6.065119	5.95	0.14
	15 **min**	7.618304	6.97811	8.215818	7.60	0.62
**Stir-frying**	5 **min**	145.9622	149.7776	148.8619	148.20	1.99
	10 **min**	125.8003	123.8813	134.041	127.91	5.40
	15 **min**	568.7039	569.2707	527.3292	555.10	24.05
**Microwaving**	3 **min**	81.09067	90.09205	91.07046	87.42	5.50
	5 **min**	100.3534	109.6569	110.8974	106.97	5.76
	7 **min**	249.4845	262.1182	270.2398	260.61	10.46
**Sous vide**	15 **min**	106.8102	113.2029	112.2244	110.75	3.44
	30 **min**	137.3397	138.3629	144.0246	139.91	3.60
	45 **min**	172.5439	176.7836	175.9064	175.08	2.24

Copper reducing antioxidant capacity absorbance values, absorbance mean and standard deviation

**Cooking Method**	**Cooking time**	**Absorbance 1**	**Absorbance 2**	**Absorbance 3**	**Absorbence mean**	**Standard deviation**
**Raw**		133.7112	138.8157	144.0511	138.86	5.17
**Boling**	5 **min**	84.55613	82.79209	82.79209	83.38	1.02
	10 **min**	104.2762	107.5384	105.7209	105.85	1.63
	15 **min**	135.4651	133.9936	133.8238	134.43	0.90
**Steaming**	5 **min**	53.71083	58.11003	57.23019	56.35	2.33
	10 **min**	8.871302	11.59442	10.68671	10.38	1.39
	15 **min**	11.50885	11.24058	11.17351	11.31	0.18
**Stir-frying**	5 **min**	450.1308	433.561	437.4855	440.39	8.66
	10 **min**	323.2737	302.1479	327.4667	317.63	13.57
	15 **min**	1436.86	1607.703	1633.613	1559.39	106.90
**Microwaving**	3 **min**	263.5	296.7659	305.4318	288.57	22.14
	5 **min**	272.1929	353.4137	344.5532	323.39	44.56
	7 **min**	735.9317	728.1968	763.4335	742.52	18.52
**Sous vide**	15 **min**	215.8317	218.6279	219.7464	218.07	2.02
	30 **min**	241.3582	238.2396	235.7058	238.43	2.83
	45 **min**	255.2177	256.4705	253.1297	254.94	1.69

2,2-diphenyl-1-picrylhydrazyl (DPPH) radical scavenging activity absorbance values, absorbance mean and standard deviation

**Cooking Method**	**Cooking time**	**IC_{50} values 1**	**IC_{50} values 2**	**IC_{50} values 3**	**IC_{50} mean**	**IC_{50} standard deviation**
**Raw**		0.238359	0.240176	0.803183	0.43	0.33
**Boling**	5 **min**	0.312792	0.335502	0.492642	0.38	0.10
	10 **min**	0.552309	0.757538	0.671654	0.66	0.10
	15 **min**	0.357477	0.427078	0.369678	0.38	0.04
**Steaming**	5 **min**	1.575335	2.100446	2.166085	1.95	0.32
	10 **min**	0.796721	0.787667	0.787667	0.79	0.01
	15 **min**	1.474781	1.506842	1.61197	1.53	0.07
**Stir-frying**	5 **min**	0.342295	0.262655	0.259606	0.29	0.05
	10 **min**	0.228686	0.184152	0.208842	0.21	0.02
	15 **min**	0.042009	0.048813	0.046834	0.05	0.00
**Microwaving**	3 **min**	0.263855	0.269707	0.238195	0.26	0.02
	5 **min**	0.452741	0.270761	0.272893	0.33	0.10
	7 **min**	0.128599	0.106474	0.095082	0.11	0.02
**Sous vide**	15 **min**	0.157892	0.148108	0.16008	0.16	0.01
	30 **min**	0.148426	0.145314	0.155414	0.15	0.01
	45 **min**	0.063943	0.070299	0.071829	0.07	0.00
