# Supplementary material for: Modern and traditional cooking methods affect the antioxidant activity and phenolic compounds content of Trachystemon Orientalis (L.) G. Don
Source: PLoS One. 2024 Feb 23;19(2):e0299037. doi: 10.1371/journal.pone.0299037 (PMC10890727; doi:10.1371/journal.pone.0299037)
Supplement: S1 Fig — (PDF) [file pone.0299037.s002.pdf]

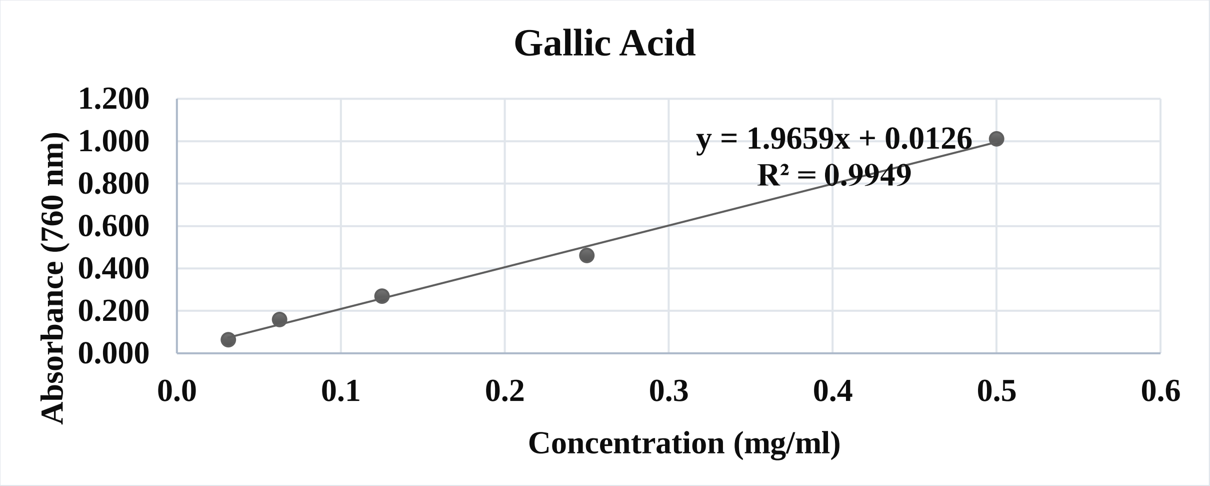

Gallic acid standard graph for the determination of total phenolic content

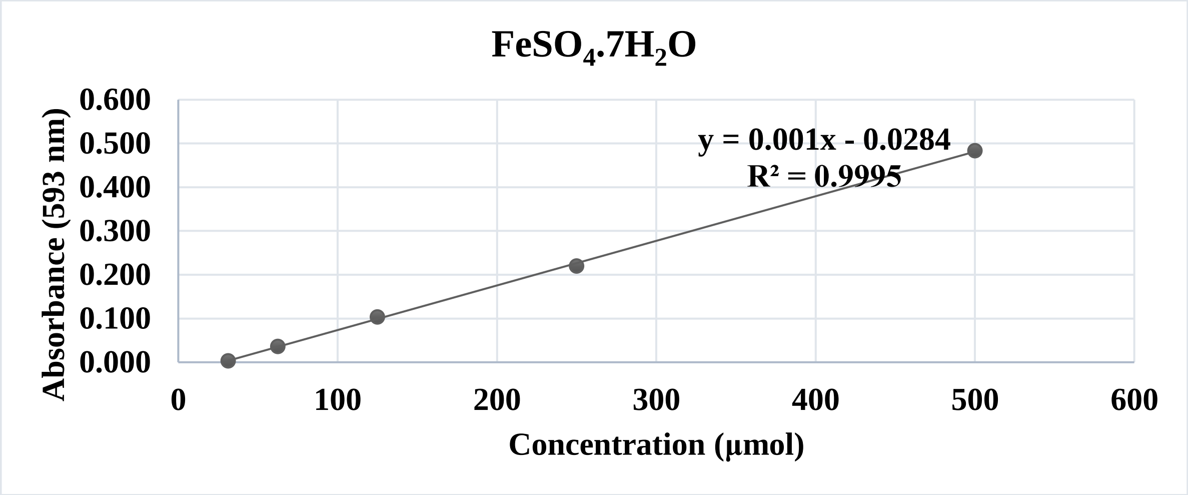

$FeSO_4.7H_2O$ standard graph for the determination of ferric Reducing antioxidant power

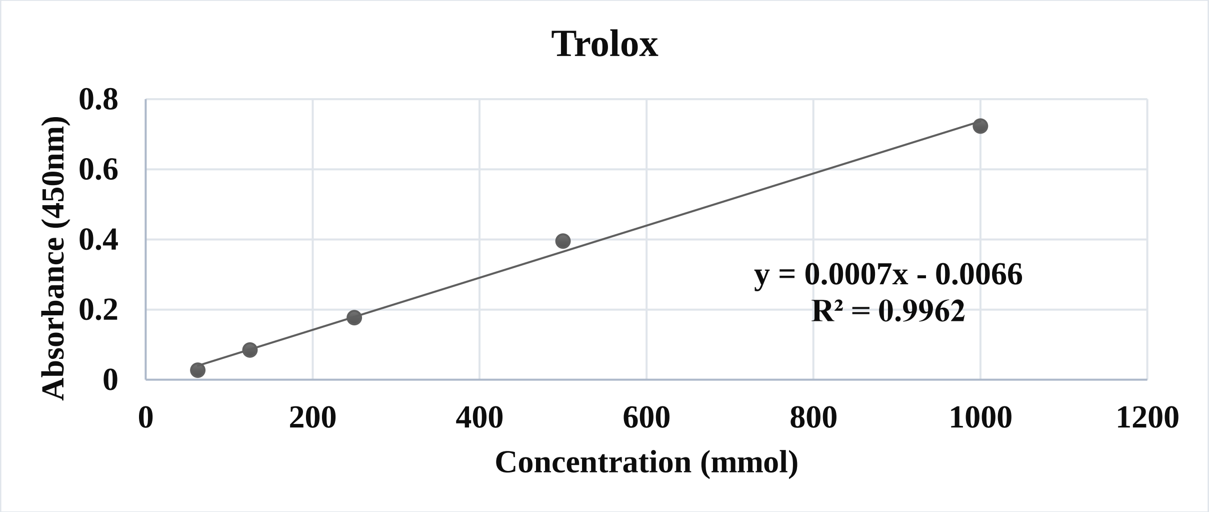

Trolox standard graph for the determination of copper reducing antioxidant capacity
