# Supplementary material for: Modern and traditional cooking methods affect the antioxidant activity and phenolic compounds content of Trachystemon Orientalis (L.) G. Don
Source: PLoS One. 2024 Feb 23;19(2):e0299037. doi: 10.1371/journal.pone.0299037 (PMC10890727; doi:10.1371/journal.pone.0299037)

280 nm chromatogram of 15 Phenolic standards mixture

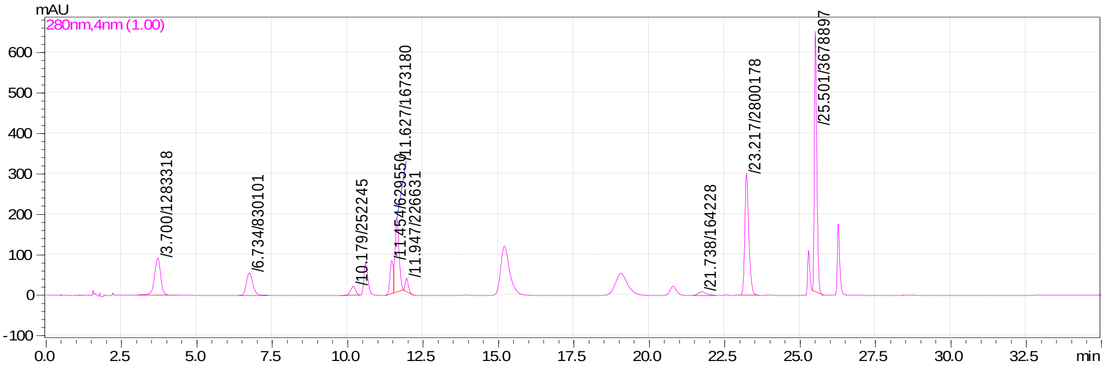

315 nm chromatogram of 15 Phenolic standards mixture

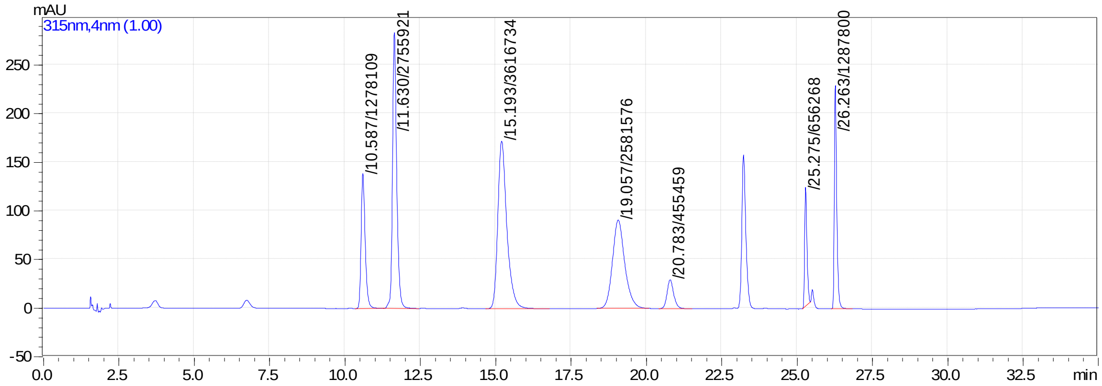

280 and 315 nm chromatograms of coded sample raw

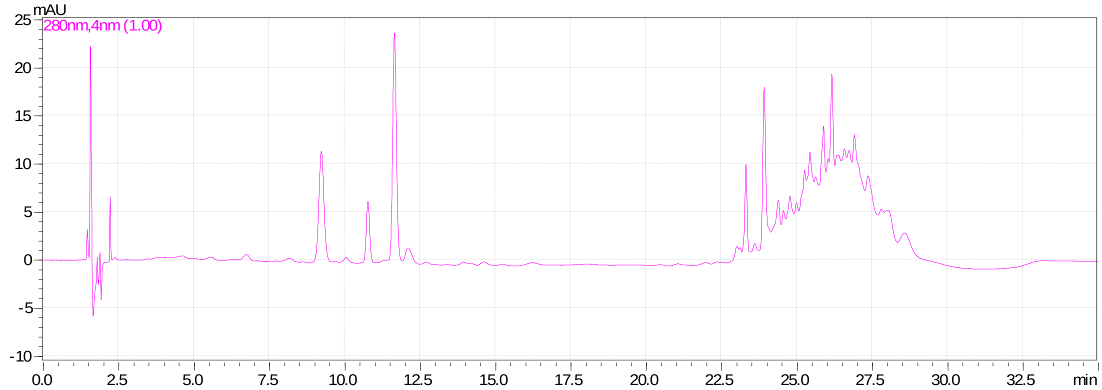

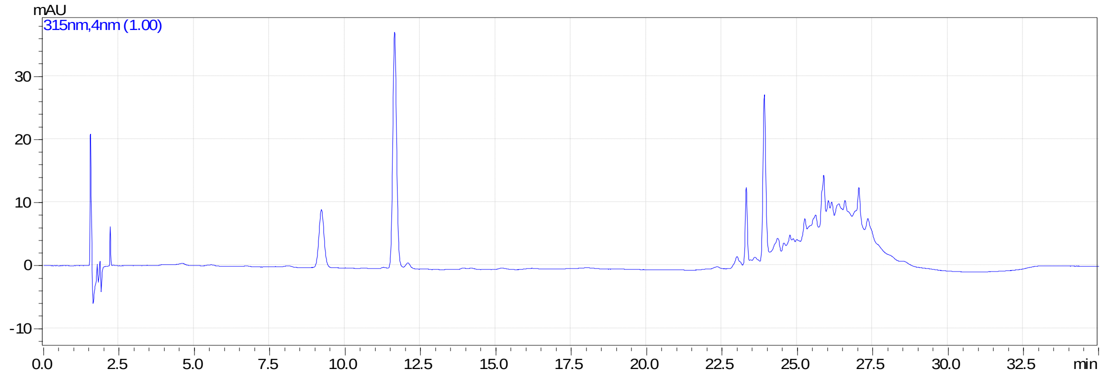

280 and 315 nm chromatograms of the sample coded Boiling-5 min

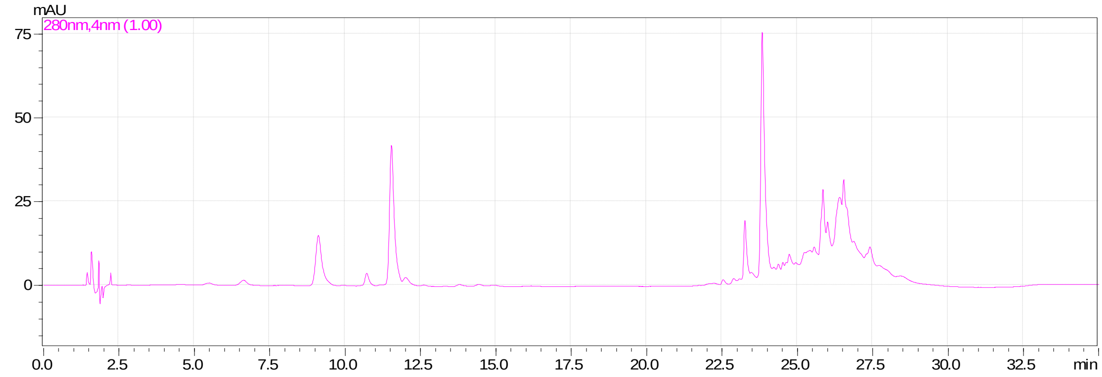

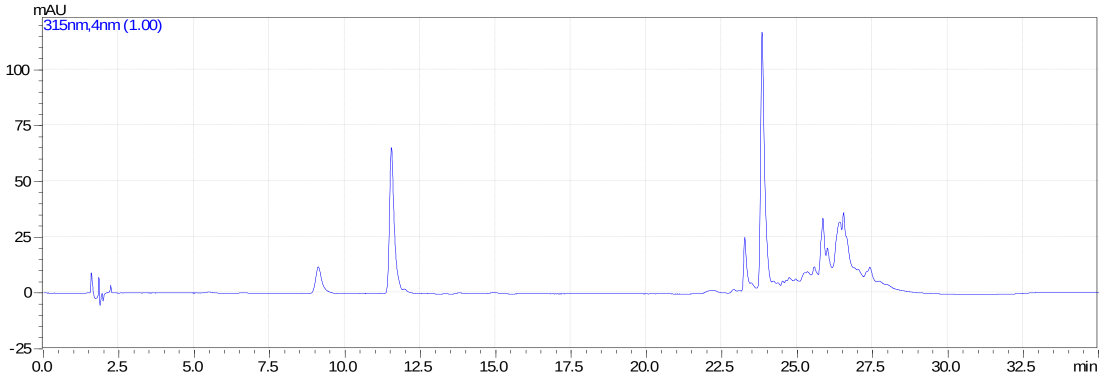

280 and 315 nm chromatograms of the sample coded Boiling- 10 min

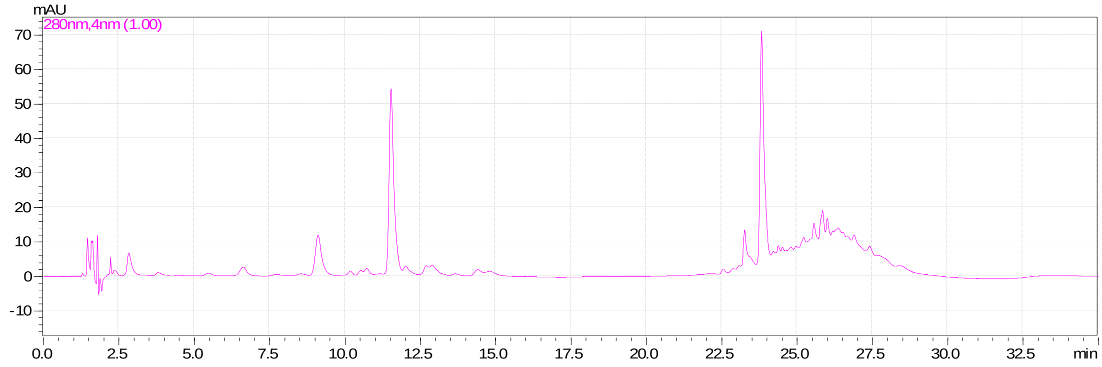

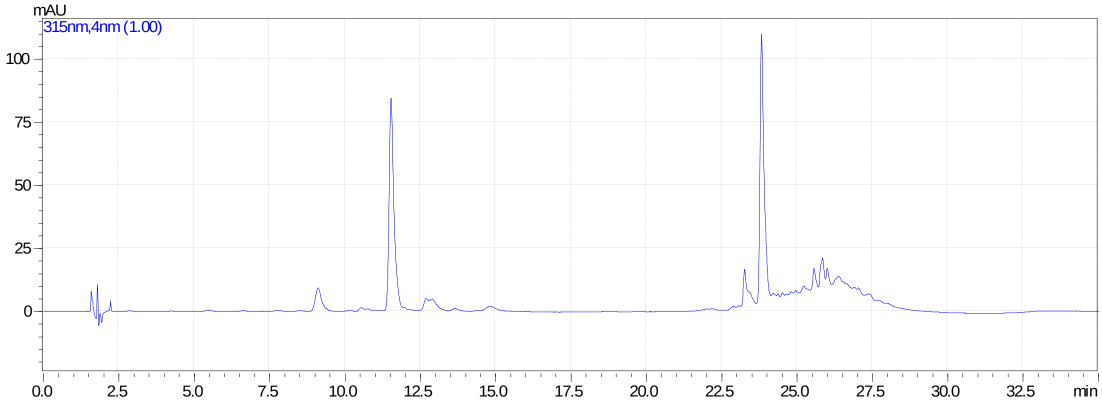

280 and 315 nm chromatograms of the sample coded Boiling -15 min

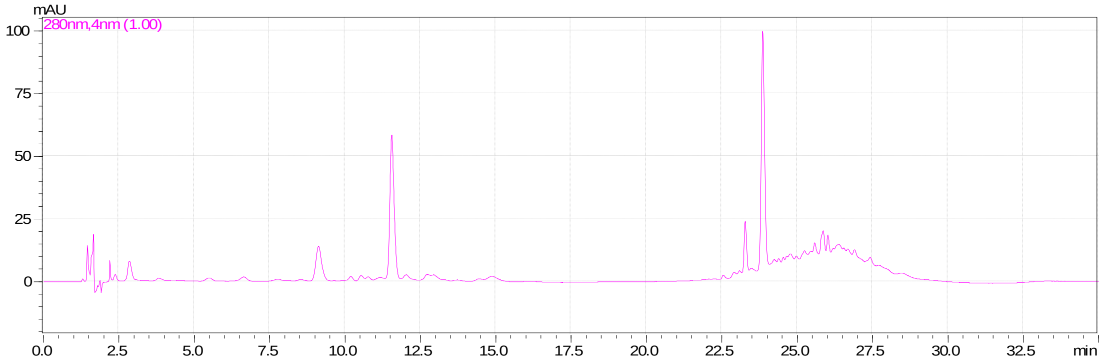

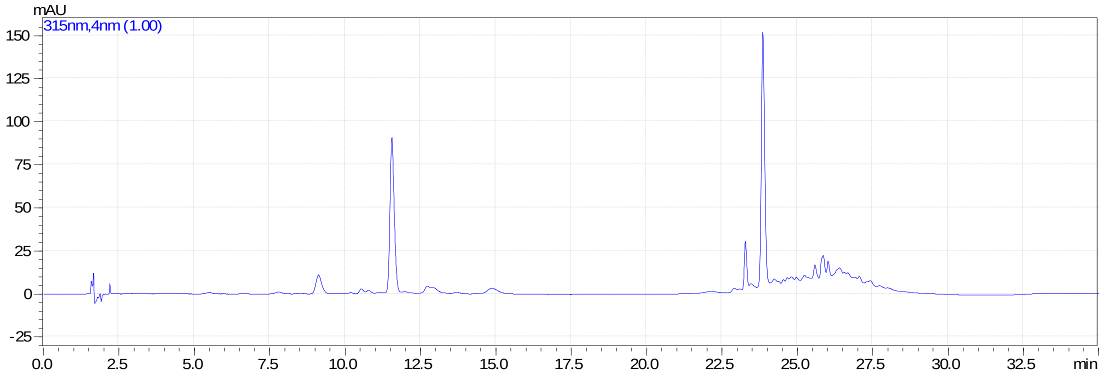

280 and 315 nm chromatograms of coded sample Steaming-5 min

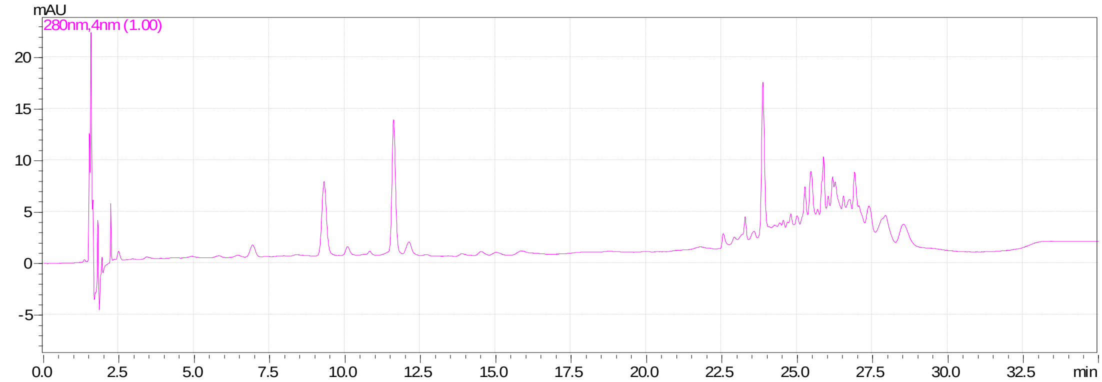

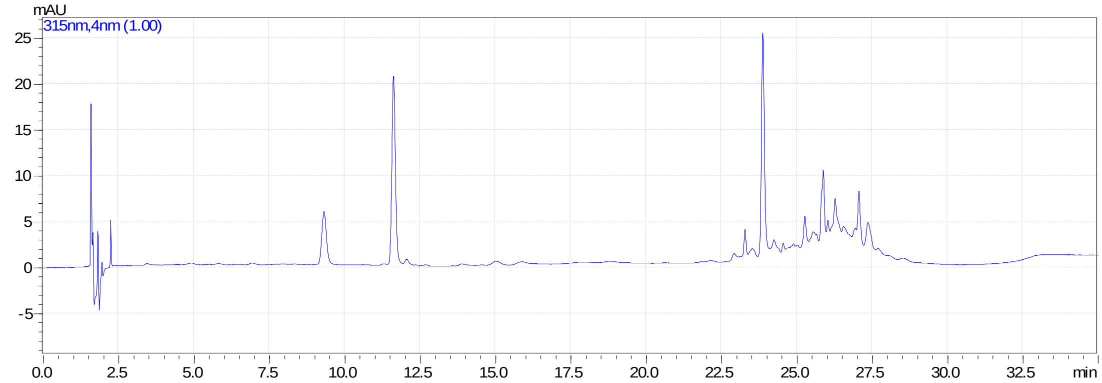

280 and 315 nm chromatograms of coded sample Steaming-10 min

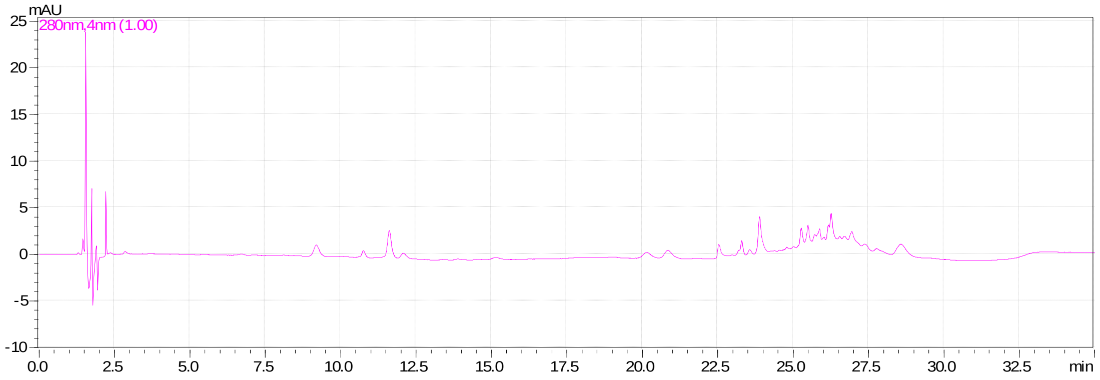

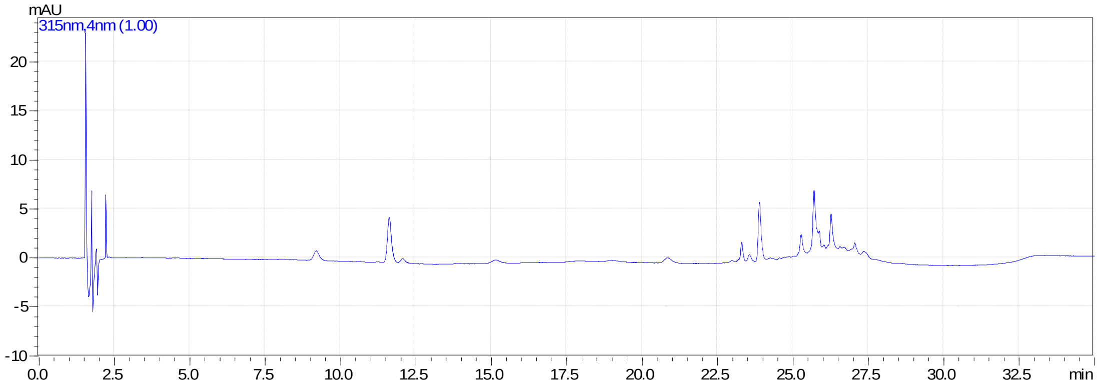

280 and 315 nm chromatograms of coded sample Steaming-15 min

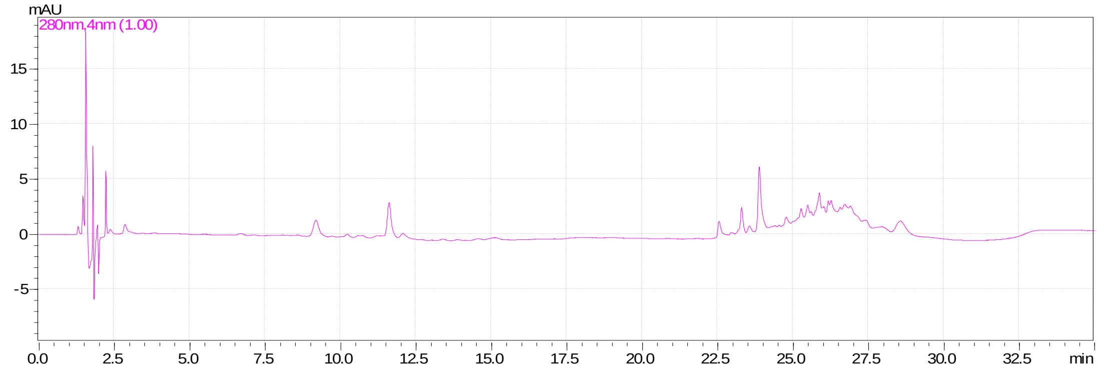

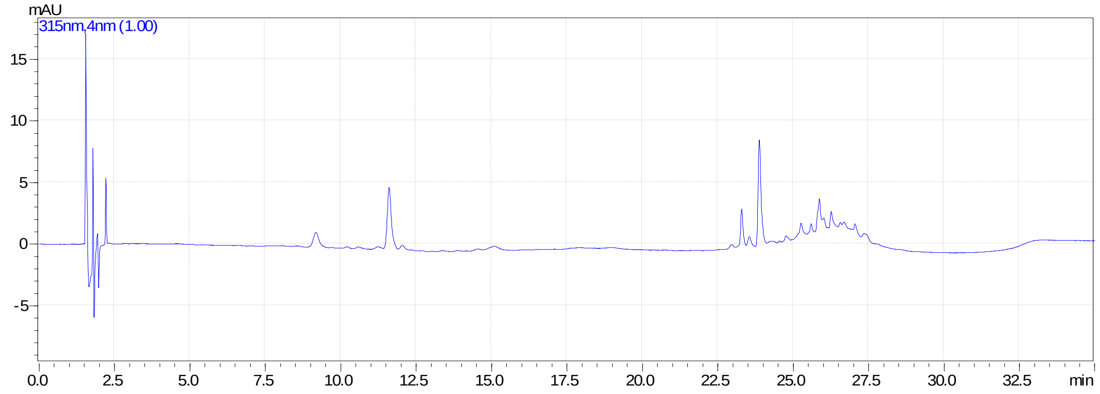

280 and 315 nm chromatograms of the sample coded Stir-frying-5 min

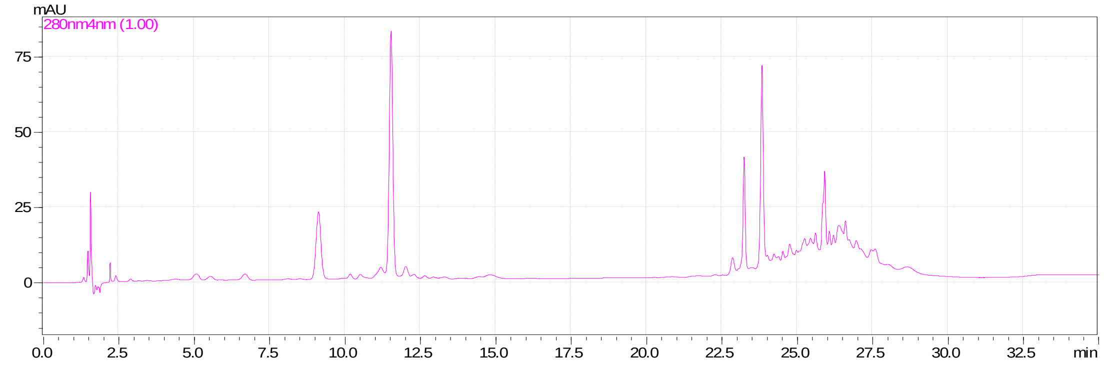

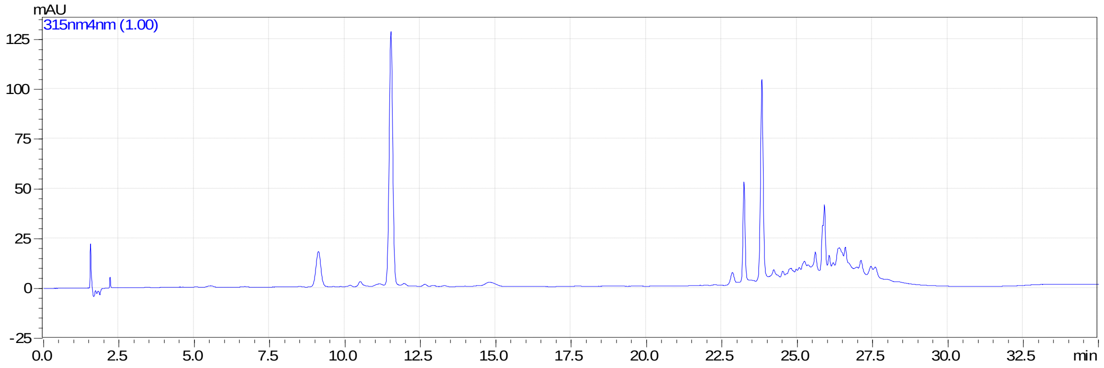

280 and 315 nm chromatograms of the sample coded Stir-frying-10 min

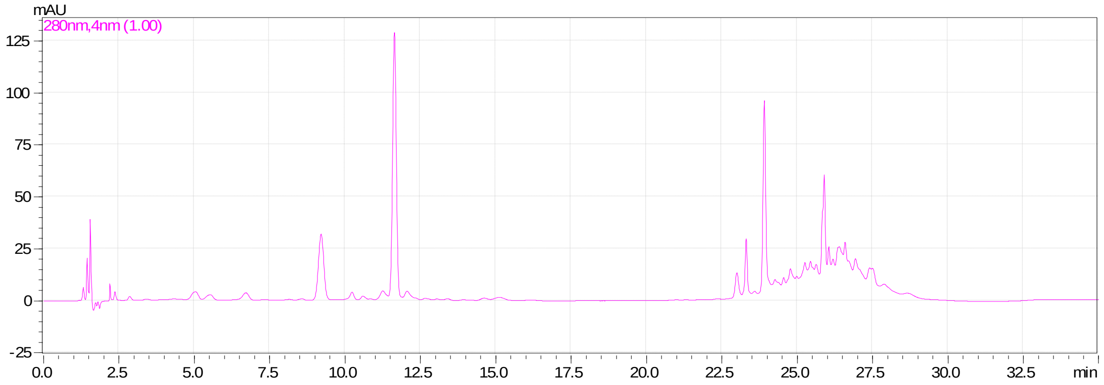

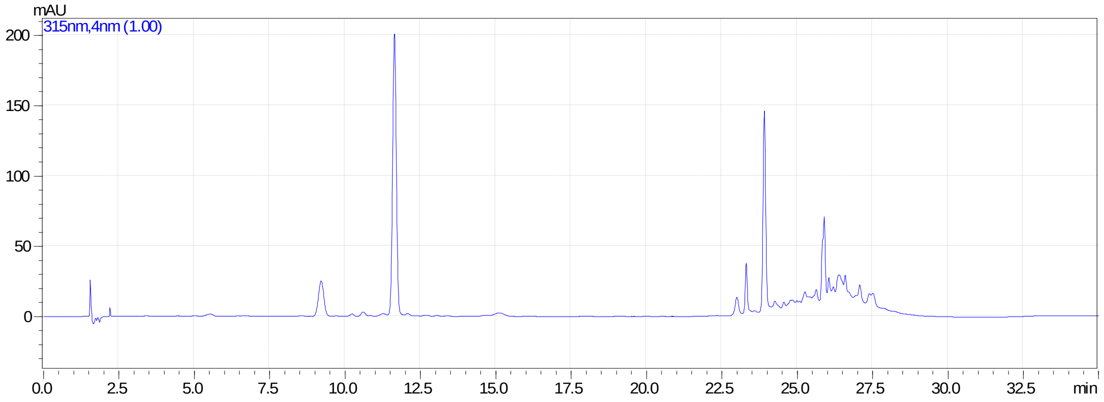

280 and 315 nm chromatograms of the sample coded Stir-frying-15 min

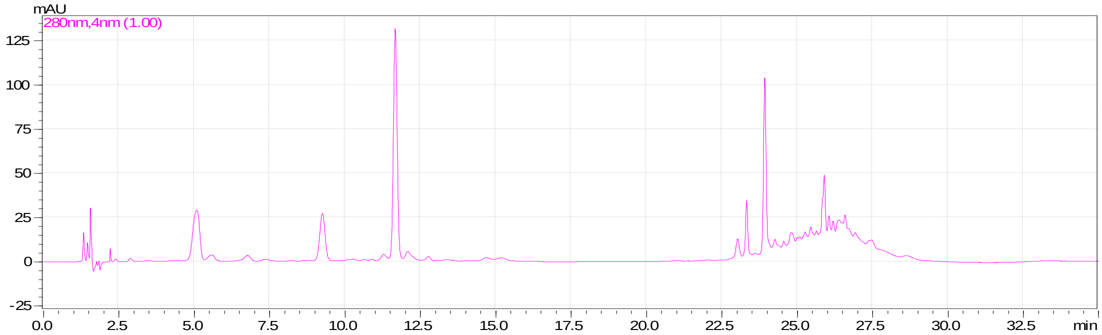

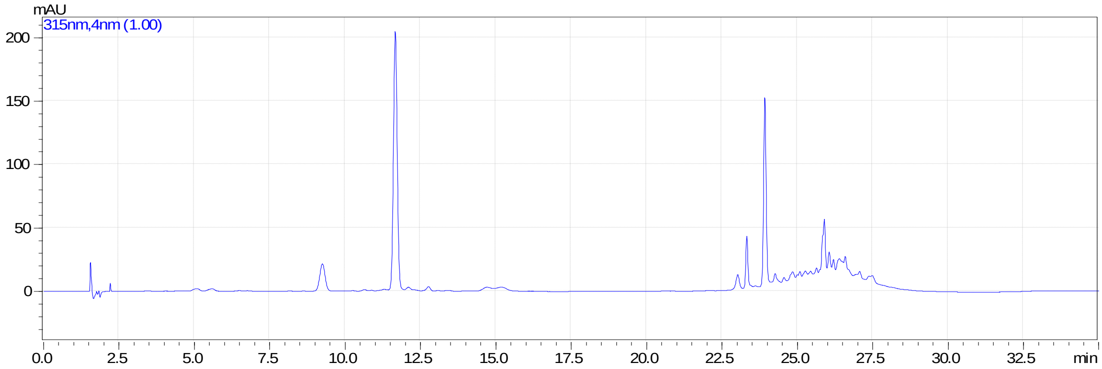

280 and 315 nm chromatograms of the sample coded Microwaving-3 min

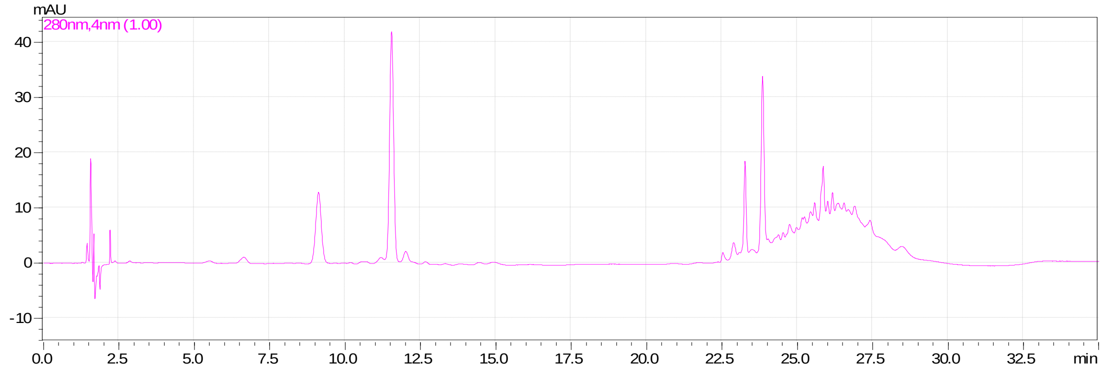

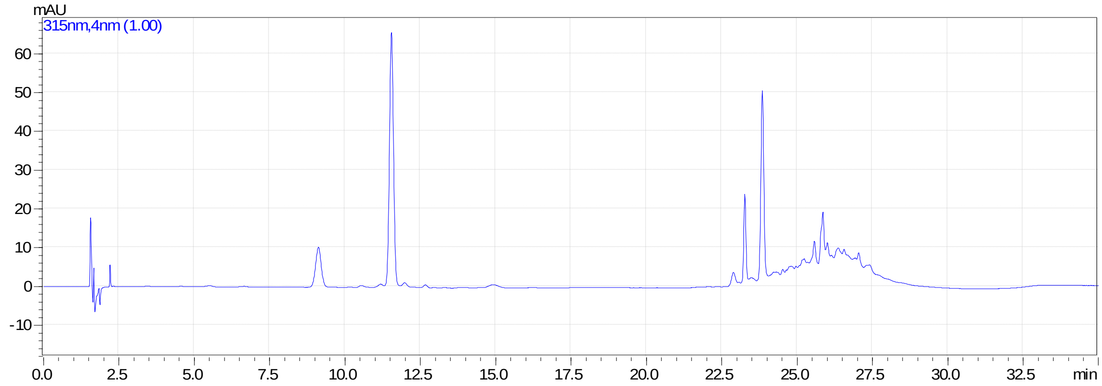

280 and 315 nm chromatograms of the sample coded Microwaving-5 min

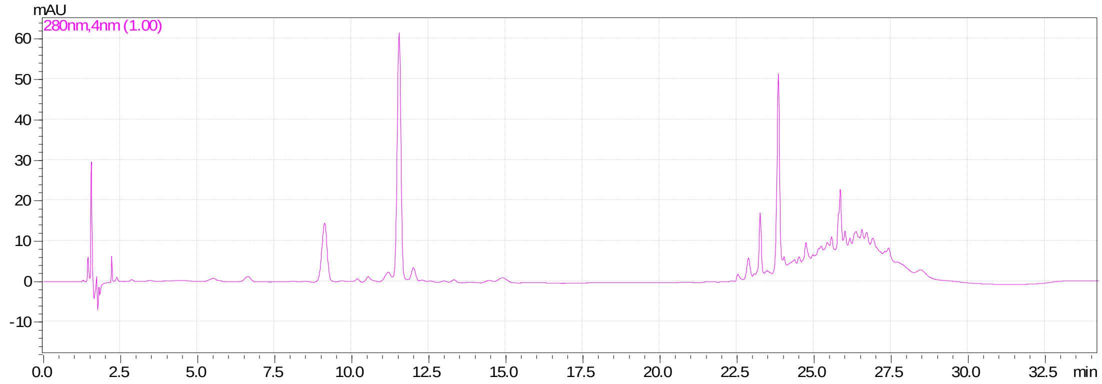

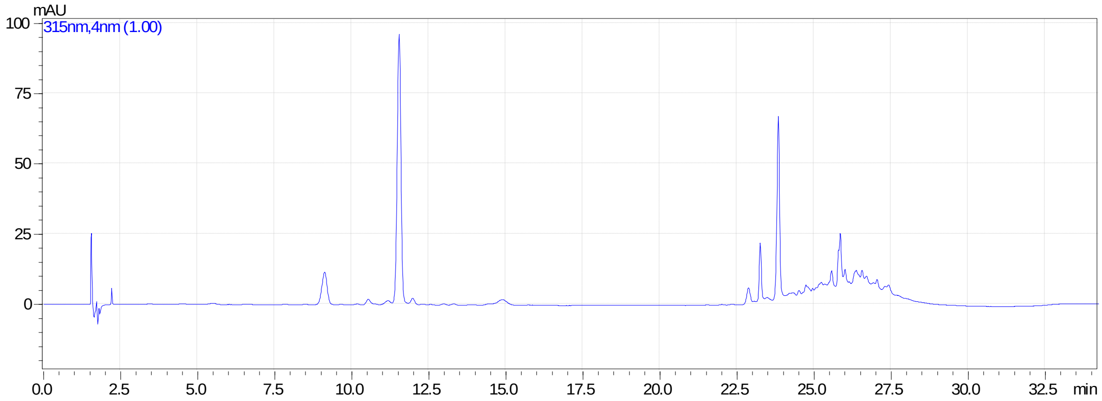

280 and 315 nm chromatograms of the sample coded Microwaving-7 min

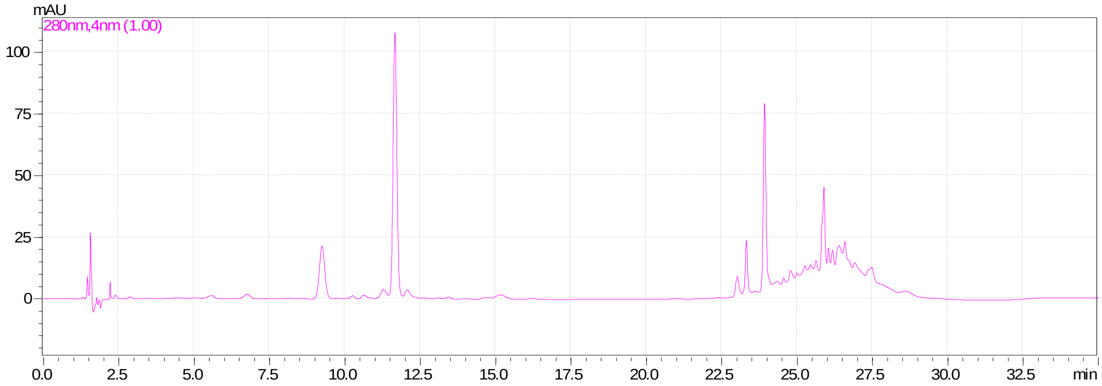

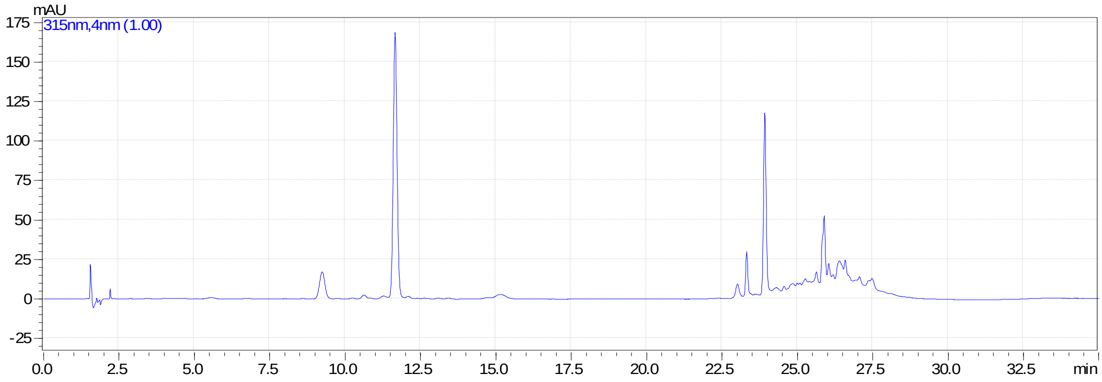

280 and 315 nm chromatograms of the sample coded Sous vide-15 min

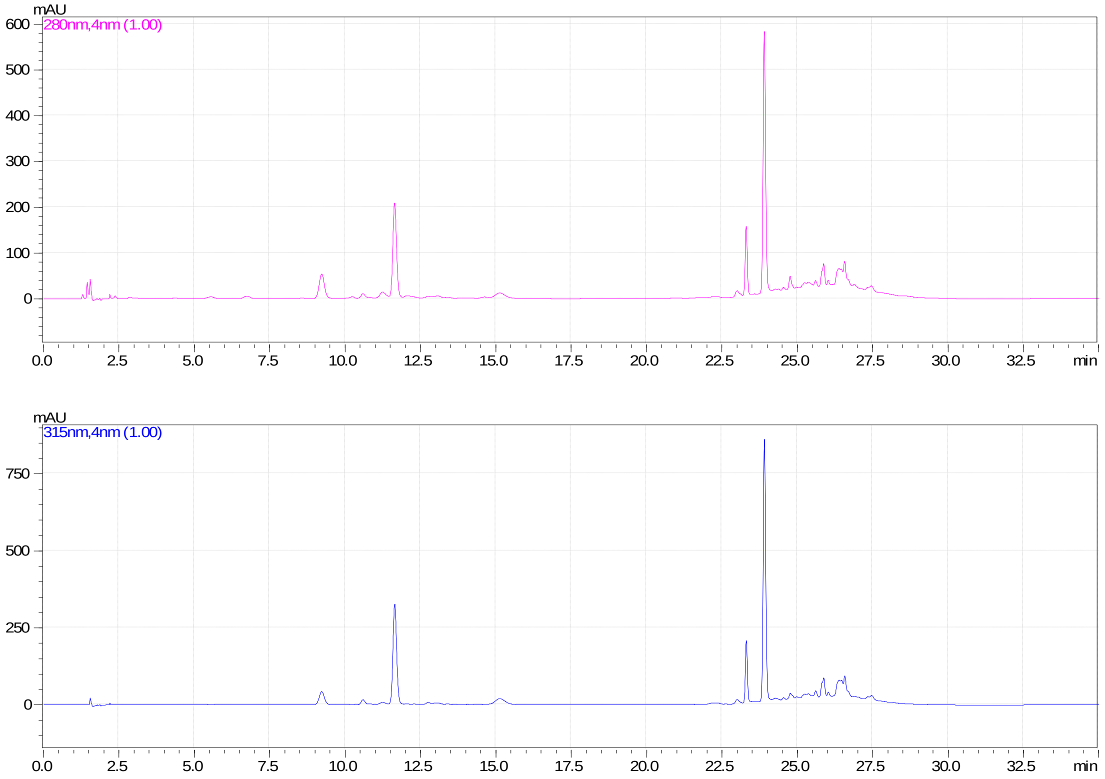

280 and 315 nm chromatograms of the sample coded Sous vide-30 min

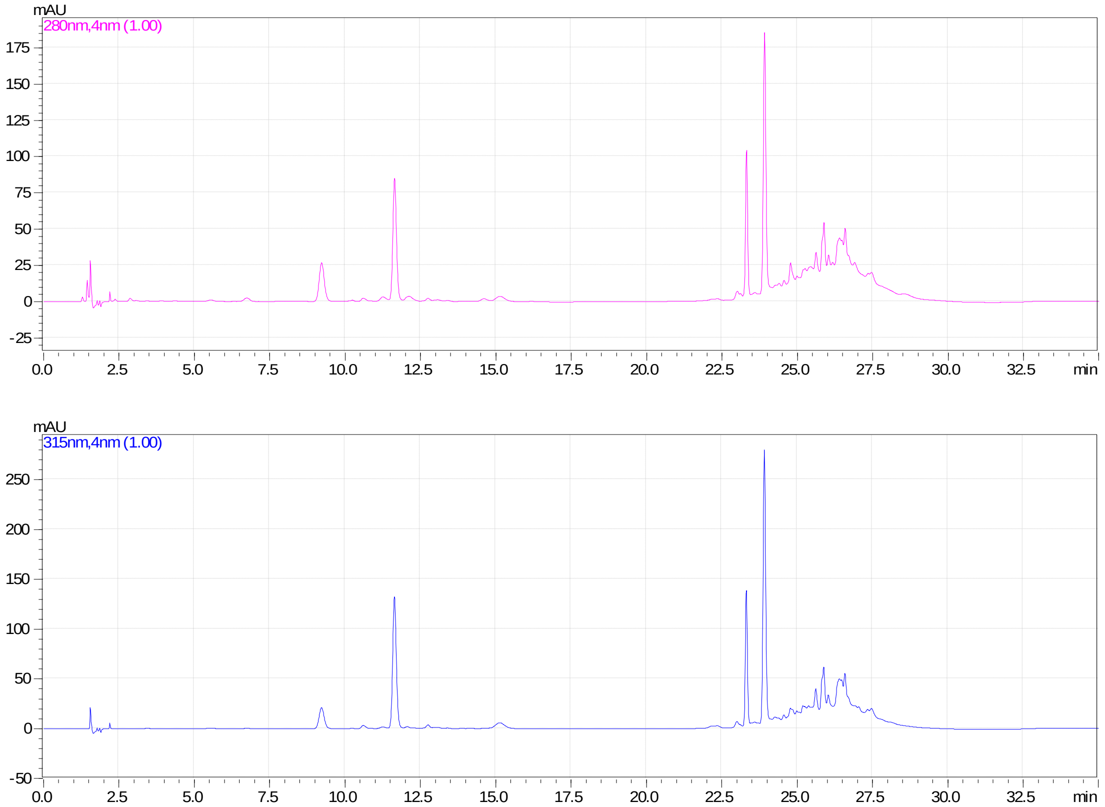

280 and 315 nm chromatograms of the sample coded Sous vide-45 min

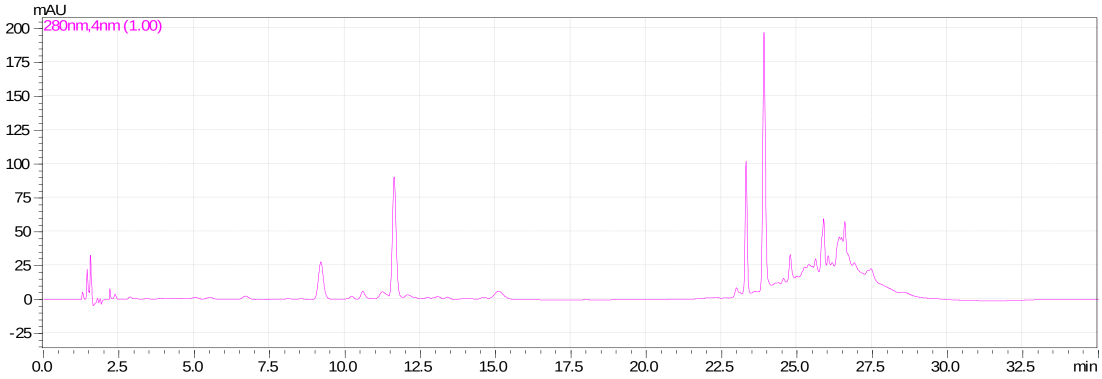

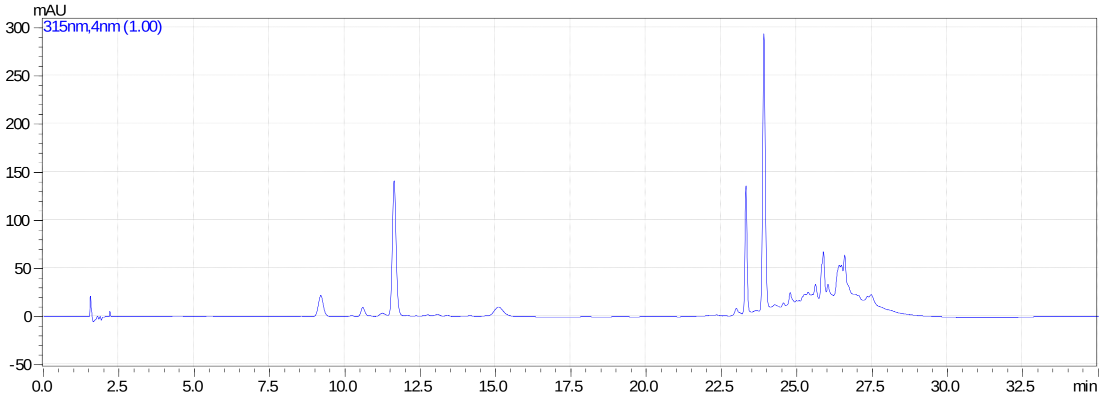

Supplement: S2 Fig — (PDF) [file pone.0299037.s003.pdf]
